# Zespoły Preekscytacji u Dzieci i młodzieży

**DOI:** 10.34763/devperiodmed.20182202.179186

**Published:** 2018-06-30

**Authors:** Aleksandra Stasiak, Katarzyna Niewiadomska-Jarosik, Piotr Kędziora

**Affiliations:** 1 Klinika Kardiologii i Reumatologii Dziecięcej II Katedra Pediatrii, Uniwersytet Medyczny w Łodzi, Polska

**Keywords:** dziecko, zespoły preekscytacji, zespół WPW, child, preexcitation syndromes, WPW syndrome

## Abstract

Zespoły preekscytacji to coraz częściej wykrywane jednostki chorobowe w populacji dzieci i młodzieży. Ich istotą jest obecność dodatkowej drogi/dróg przewodzenia w sercu, przez którą impuls przewodzony jest szybciej niż fizjologicznie. Prowadzi to do szybszego pobudzenia komór i może być podłożem powstawania groźnych arytmii. Najczęstszym zespołem preekscytacji jest zespół Wolffa-Parkinsona--White’a, który dotyczy do 2/1000 osób. Obecność dodatkowej drogi przewodzenia może skutkować wystąpieniem poważnych konsekwencji, poczynając od częstoskurczu nadkomorowego, a kończąc na nagłym zgonie sercowym. Istnieją zarówno inwazyjne, jak i nieinwazyjne metody diagnostyczne zespołów preekscytacji. Postępowanie terapeutyczne obejmuje farmakoterapię oraz zabieg przerwania ciągłości dodatkowej drogi przewodzenia zwany ablacją, co pozwala trwale usunąć przyczynę arytmii.

## Abstract (en)

Preexcitation syndromes are becoming a more often detected disease in the pediatric population. Their essential feature is the presence of additional paths / pathways in the heart, through which the impulse is conducted faster than physiologically. This leads to faster ventricular excitation and may lead to creating dangerous arrhythmias. The most common preexcitation syndrome is Wolff - Parkinson-White syndrome, which affects 2/ 1000 people. The presence of an accessory pathway could result in serious consequences, ranging from supraventricular tachycardia to sudden cardiac death. There are both invasive and non-invasive methods of diagnosing preexcitation syndromes. Therapeutic treatment includes pharmacotherapy and ablation, which makes it possible to permanently remove the cause of the arrhythmia.

## Etiologia

Zespoły preekscytacji definiowane są, jako jednostki chorobowe, w których dochodzi do przedwczesnej aktywacji miokardium komór przez impuls, który jest przewodzony przez nieprawidłową drogę i omija fizjologiczne opóźnienie w węźle przedsionkowo-komorowym. Zostało opisanych kilka typów zespołów preekscytacji, a różni je od siebie anatomia nieprawidłowej dodatkowej drogi przewodzenia [1]. W zespołach preekscytacji istnieją nieprawidłowe połączenia przewodzące impulsy z przedsionków do komór, które mogą występować pomiędzy przedsionkiem a komorą, pomiędzy węzłem przedsionkowo-komorowym a osią His - Purkinje i inne. Dodatkowa droga przewodzi impulsy z przedsionków do komór szybciej niż węzeł przedsionkowo-komorowy. Przewodząc pierwotny impuls, potencjalnie stwarza warunki do przedwczesnego pobudzenia komór, co prowadzi do nawracających arytmii i napadowych częstoskurczów przedsionkowo - komorowych. Impuls przewodzony jest szybciej dodatkową drogą przedsionkowo-komorową, zanim oś węzeł przedsionkowo komorowy – pęczek Hisa rozpocznie swą depolaryzację [1]. Mechanizm, w jakim impuls przedwcześnie pobudza komory to tzw. zjawisko reentry (rycina 1). Jest to zaburzenie, w którym impuls ponownie stymuluje tkankę już wcześniej depolaryzowaną. Do zaistnienia mechanizmu reentry konieczne jest istnienie dodatkowej drogi przewodzenia, istnienie bloku w części tej drogi oraz opóźnienie przewodzenia w pozostałej części drogi. W mechanizmie reentry impulsy elektryczne są opóźniane lub blokowane w jednej lub kilku strefach układu przewodzącego serca w czasie prawidłowego przewodzenia przez pozostałą część układu przewodzącego. W wyniku tego mechanizmu opóźnione bodźce elektryczne ponownie stymulują komórki wcześniej już depolaryzowane przez bodźce przewodzone prawidłowo. Jeżeli opóźnione impulsy stymulują strefę znajdującą się w okresie względnej refrakcji, mogą wyzwolić ponowne pobudzenie. Taki mechanizm wywołuje pojedyncze pobudzenie przedwczesne lub powtarzające się pobudzenia. Do zaburzeń rytmu powstających w mechanizmie reentry w preekscytacji zalicza się nawrotny częstoskurcz przedsionkowo-komorowy (AVRT – atrioventricular reentrant tachycardia) [2].

**Ryc. 1:**
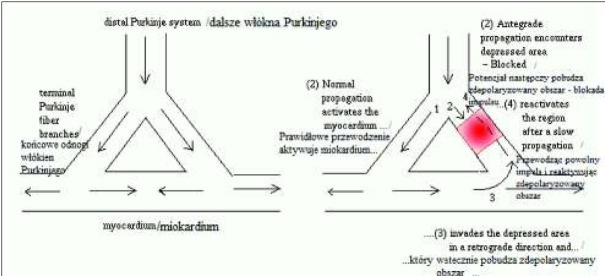
Schemat mechanizmu reentry. Po lewej stronie prawidłowe przewodzenie impulsu w sercu, po prawej stronie zjawisko reentry. **[**Arnold M. *Spatiotemporal dynamics of termination by pacing*. A model of reentry [image on the Internet] [stan z 11.02.2014]: http://www.imsc.res.in/~sitabhra/research/heart/pacing/] Fig. 1. A diagram showing mechanism of reentry. On the left side correct conduction of an impulse, on the right side reentry.

## Podział

Częstość występowania zespołów preekscytacji u dzieci wynosi od 1:250 do 1:1000 [3]. Wyróżniamy trzy podstawowe zespoły preekscytacji: zespół Wolffa-Parkinsona-White’a (WPW), zespół Lowna-Ganonga--Levine’a (LGL) oraz typ Mahaima. Trzy główne zespoły preekscytacji różnią się od siebie cechami zapisu EKG oraz przewodzeniem dodatkowego pobudzenia. Preekscytacja o typie WPW cechuje się krótkim odstępem PR, obecnością fali delta oraz poszerzonym zespołem QRS, a dodatkowe pobudzenie przewodzone jest przez pęczek Kenta z pominięciem węzła przedsionkowo-komorowego. W zespole Lowna-Ganonga-Levine’a odstęp PR jest skrócony, natomiast brak jest fali delta i nieprawidłowego zespołu QRS (rycina 2). Typ Mahaima natomiast charakteryzuje się przewodzeniem impulsu przez włókna Mahaima i cechuje się prawidłowym odstępem PR, obecnością fali delta oraz poszerzonym zespołem QRS [4].

**Ryc. 2:**
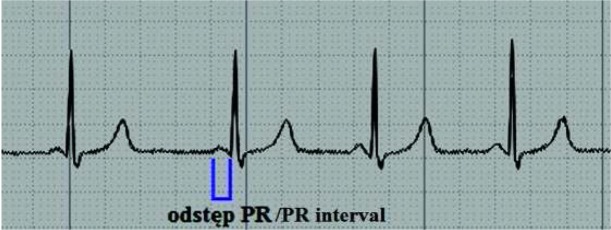
Fragment zapisu EKG przedstawiający skrócony odstęp PR w zespole LGL. [*Sindrome de Wolff-Parkinson-White*. [image on the Internet] .[stan z 12.03.2014] http://www.my-ekg.com/arritmias-cardiacas/sindromes-preexcitacion.html] Fig. 2. A fragment of ECG showing a shortened PR interval in LGL syndrome.

Najczęściej spotykaną postacią preekscytacji występującą u dzieci jest typ Wolffa-Parkinsona-White’a [5]. Ten typ preekscytacji określany jest, jako wrodzona anomalia związana z obecnością nieprawidłowej tkanki przewodzącej pomiędzy przedsionkami i komorami, która stanowi drogę dla nawrotnego częstoskurczu. Wyróżniamy trzy typy WPW: jawny, utajony oraz intermitujący. Jawny zespół WPW to taki, w którym typowe cechy preekscytacji występują w spoczynkowym zapisie EKG stale. Utajony zespół WPW to taki, w którym cechy preekscytacji występują w zapisie EKG tylko w trakcie napadu częstoskurczu nadkomorowego, natomiast intermitujący zespół WPW to taki, w którym cechy preekscytacji nie są widoczne na stałe w zapisie EKG, a pojawiają się w pewnych okolicznościach (np. w trakcie wysiłku fizycznego). Częstość występowania preekscytacji typu Wolffa-Parkinsona-White’a w dzieciństwie szacuje się na 0,4-2,2 na 1000 osób, przeważa płeć męska [6]. Szacuje się, że od 20-37% niemowląt z WPW ma inne współtowarzyszące wady wrodzone serca [6]. Najczęściej spotykaną wadą wrodzoną u pacjentów z zespołem WPW jest anomalia Ebsteine’a, w której dodatkowe drogi przewodzenia są niemal zawsze prawostronne. Pozostałe nieprawidłowości anatomiczne, w których częściej występuje preekscytacja to: wypadanie płatków zastawki mitralnej, ubytek przegrody międzyprzedsionkowej, transpozycja wielkich naczyń, koarktacja aorty, dekstrokardia, ubytek przegrody międzykomorowej, guzy serca (rhabdomyoma), zespół Marfana oraz kardiomiopatia przerostowa [7]. Krewni pierwszego stopnia pacjentów dotkniętych tym zespołem posiadają zwiększone ryzyko wystąpienia preekscytacji, które szacowane jest na 5,5 na 1000 osób [6]. Ostatnie badania genetyczne pozwoliły na odkrycie rodzinnej postaci preekscytacji o typie WPW dziedziczącej się w sposób autosomalny dominujący, związanej z genem znajdującym się na długim ramieniu chromosomu 7. Z badań wynika, że 3% chorych z zespołem WPW posiada objawowych krewnych pierwszego stopnia [1].

Istnieją trzy główne kryteria, które muszą zostać spełnione, aby potwierdzić preekscytację o typie WPW w 12-odprowadzeniowym zapisie EKG (rycina 3). Są to:

**Ryc. 3:**
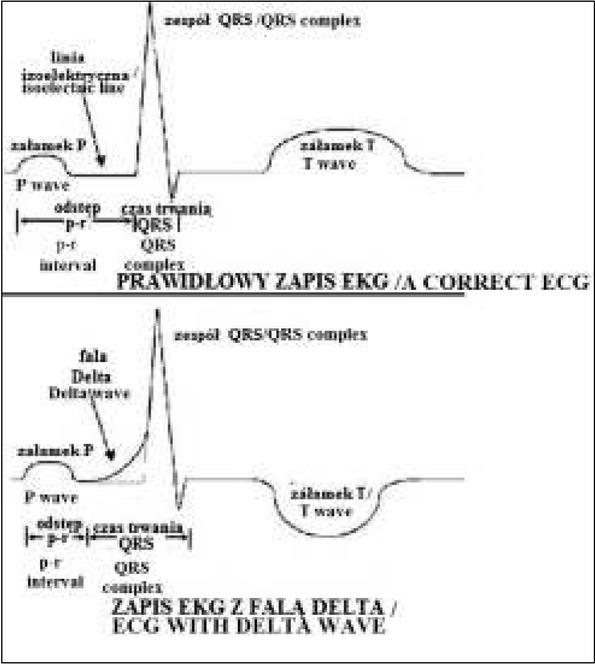
Schemat przedstawiający prawidłowy zapis EKG oraz zapis EKG zespołu WPW. [*Ventricular pre-excitation: WPW and LGL syndromes*. [image on the Internet]. 2006 Aug 18 [stan z 12.03.2014] http://www.frca.co.uk/article.aspx?articleid=100691#faq] Fig. 3. A diagram showing a regular ECG record and a record of WPW syndrome.

- krotki odstep PR (ponizej) dolnej granicy normy odpowiednio do wieku pacjenta – 0,08 s poniżej 3 r.ż., 0,1 s do 16 r.ż. i 0,12s powyżej 16 r.ż.) manifestujący wcześniejszą depolaryzację komór [1]
- obecna fala delta na ramieniu wstepującym załamkaR reprezentująca stosunkowo chaotyczną i powolną depolaryzację komór poprzez dodatkową drogę przedsionkowo-komorową [1]
- poszerzony zespoł QRS (za wydłuzony uznaje sie u dzieci poniżej 4 r.ż. czas trwania zespołu QRS powyżej 90 ms, u starszych w wieku 4–16 lat powyżej 100 ms) [8, 9].

Dodatkowo mogą być obecne zaburzenia repolaryzacji manifestujące się zmianą odchylenia fali ST-T w przeciwną stronę niż wektor zespołu QRS [1, 8].

Badanie elektrokardiograftczne pozwala na zlokalizowanie dodatkowej drogi przewodzenia w 1 z 5 głównych położeń: po lewej stronie serca u około 50% pacjentów, po prawej stronie serca u 20%, w regionie przegrody u około 30% pacjentów, a ok. 7% pacjentów ma wiele połączeń dodatkowych [6, 7].

## Objawy

U pacjentów z cechami preekscytacji w EKG tylko ok. 50% wykazuje jakiekolwiek objawy, z czego najczęstszym jest kołatanie serca, które jest manifestacją nawrotnego częstoskurczu przedsionkowo-komorowego (AVRT). Młodsze dzieci opisują ten objaw, jako ból w klatce piersiowej, pulsowanie w gardle, a nawet ból brzucha. Może wystąpić również zasłabnięcie czy omdlenie. Następstwem utrwalonego AVRT może być migotanie przedsionków, a także zatrzymanie krążenia w mechanizmie migotania komór. Niemowlęta z AVRT mogą prezentować niespecyficzne objawy, takie jak drażliwość, niechęć do jedzenia, objawy ze strony przewodu pokarmowego, dróg oddechowych, niepokój, a nawet wstrząs. Objawy podczas częstoskurczu różnią się w zależności od wieku, szybkości częstoskurczu oraz współistniejących chorób serca [1].

Wyróżniamy podział na dwie grupy tachyarytmii występujące w przebiegu zespołu WPW ze względu na to czy dodatkowa droga przewodzenia jest integralną częścią pętli reentry czy też nie. W pierwszym przypadku mamy do czynienia z częstoskurczem ortodromowym lub antydromowym, natomiast w drugim jest to najczęściej tachyarytmia przedsionkowa oraz częstoskurcz komorowy. Podczas częstoskurczu ortodromowego pętlę reentry tworzy: mięsień przedsionków, układ bodźcoprzewodzący, dodatkowa droga przewodzenia (pobudzana wstecznie) oraz mięsień komór. W tym przypadku zespoły QRS są najczęściej wąskie. Natomiast w częstoskurczu antydromowym jest przewodzenie przez dodatkową drogę w kierunku zstępującym i wsteczna aktywacja układu bodźco-przewodzącego z pobudzeniem przedsionków. W tym przypadku uwidacznia się maksymalna preekscytacja komór, co w obrazie EKG daje szerokie zespoły QRS [10] (rycina 4).

**Ryc. 4:**
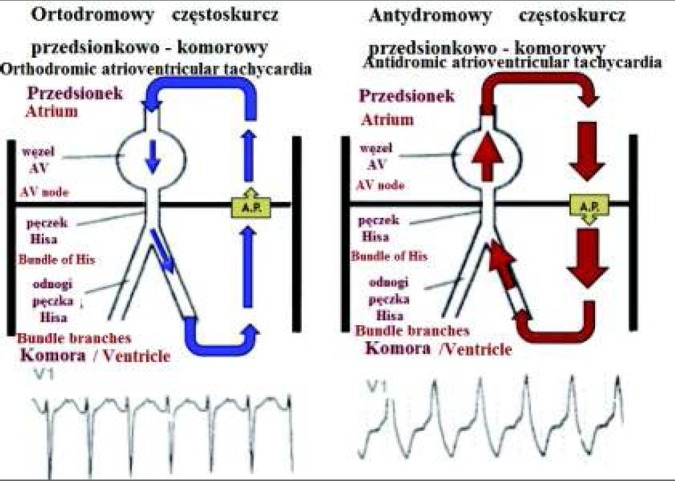
Po lewej stronie częstoskurcz ortodromowy z wąskim zespołem QRS, po prawej stronie częstoskurcz antydromowy z szerokim zespołem QRS. [*WPW & AV reciprocating tachycardia*. [image on the Internet]. [Cite 2014 Feb 11] http://www.permanente.net/homepage/kaiser/pages/f63698.html] Fig. 4. On the left side orthodromic tachycardia with a narrow QRS complex, on the right side antidromic tachycardia with wide QRS complex.

Preekscytacja jest relatywnie częstą przyczyną częstoskurczu nadkomorowego. Około 60% dzieci z AVRT wykazuje objawy arytmii w pierwszym roku życia, najczęściej w 3-4 miesiącu życia. U ponad 20% nowo zdiagnozowanych dzieci z AVRT stwierdza się w EKG zespół WPW po przywróceniu rytmu zatokowego. Wiele z tych dzieci wraz z wiekiem traci predyspozycje do częstoskurczu w wyniku zmiany właściwości elektrycznych dodatkowych dróg przewodzenia [1]. Ujawnieniu się częstoskurczu sprzyjają infekcje, wysiłek fizyczny, a także okres dojrzewania, dlatego często wykrywane są one u młodzieży. Nagły zgon u pacjentów z zespołem preekscytacji może nastąpić na skutek migotania przedsionków, które może prowadzić do migotania komór [11]. W pracy Orczykowskiego i wsp. ryzyko wystąpienia nagłego zgonu sercowego u pacjentów z zespołem WPW określono na 0,15%-0,39% [12]. Z badanych 60 bezobjawowych dzieci u 6 (10%) objaw zagrażający życiu (migotanie komór, zatrzymanie krążenia) okazał się pierwszą oznaką choroby. U 48% z 42 pediatrycznych pacjentów z dodatkowymi drogami przewodzenia zatrzymanie krążenia było pierwszym objawem choroby. W niedawno przeprowadzonym prospektywnym badaniu wykazano, że 1,6% ze 184 przebadanych dzieci z zespołem WPW przebyło zatrzymanie krążenia pod postacią migotania komór, które zostało skutecznie zresuscytowane. Migotanie komór odnotowano u 2,2% objawowych pacjentów z WPW. U niektórych pacjentów migotanie komór było pierwszą manifestacją tej choroby. Autorzy wyciągnęli też wniosek, że pacjenci z intermitującym zespołem WPW na ogół posiadają słabe przewodzenie antydromowe, co niesie za sobą niższe ryzyko nagłego zgonu [13]. W badaniach przeprowadzanych na grupie bezobjawowych pacjentów pediatrycznych z zespołem WPW w wieku 8-12 lat, u których co 6 miesięcy przeprowadzano kontrolne EKG oraz 24-godzinne zapisy metodą Holtera po 57 miesiącach badań 51 ze 133 dzieci rozwinęło objawowy zespół, a 19 z nich rozwinęło potencjalnie zagrażającą życiu arytmie. Co ciekawe, niektóre dzieci wykazywały atypowe objawy, jak np. nudności, nagłą męczliwość, ból brzucha, niechęć do jedzenia czy niemożność koncentracji w trakcie zabawy [14].

Cechy zapisu EKG, które mogą sugerować wystąpienie zespołu WPW, czyli najczęstszej formy zespołu preekscytacji, w analizie 12 odprowadzeniowego EKG można znaleźć u 1,5-3,1 na 1000 osób. Rzeczywista częstość występowania zespołu WPW może być większa, ponieważ nie we wszystkich przypadkach cechy preekscytacji widoczne są na standardowym 12-odprowadzeniowym zapisie EKG, w momencie, gdy nie występuje napadowy częstoskurcz [1].

## Metody diagnostyczne

Do metod diagnostycznych u pacjentów z zespołami preekscytacji zalicza się:

- EKG: ocena rodzaju zespołu preekscytacji.
- Holter EKG: ocenia nasilenie zaburzen rytmu orazumożliwia wykrycie utajonej/intermitującej postaci zespołu WPW, która nie zawsze jest uchwytna w momencie wykonywania standardowego EKG.
- Badanie echokardiograficzne: ocenia anotomie serca (współistnienie towarzyszących wad serca) oraz funkcję lewej komory.
- Proba wysiłkowa: nieinwazyjny i prosty w wykonaniu test. Nagły zanik fali delta podczas próby wysiłkowej koreluje z następowym efektywnym okresem refrakcji dodatkowej drogi (anterograde effective *refractory period* of accessory pathway -APERP). Wadą tego testu jest fakt, iż potrafi on całkowicie wykluczyć ryzyko nagłego zgonu tylko u 8% pacjentów zarówno dzieci, jak i dorosłych [11].
- Badania elektrofizjologiczne: okreslają ryzyko nagłegozgonu sercowego poprzez pomiar najkrótszego przedwczesnego odstępu R-R podczas migotania przedsionków. Jeśli wynosi on poniżej 250 ms oznacza to duże ryzyko nagłego zgonu, jeśli zaś powyżej 250 ms to oznacza to brak zagrożenia życia dla dziecka [11].
- Istnieją rowniez rzadziej wykonywane inwazyjne metody wykrycia zespołów preekscytacji, które są szczególnie przydatne w utajonych zespołach oraz przydatne w mapowaniu dodatkowej drogi przed zabiegiem ablacji. Są to:
- Przezprzełykowa stymulacja serca: jest to minimalnieinwazyjna i generalnie łatwa do przeprowadzenia procedura. Specjalny cewnik z elektrodą wprowadzany jest przez jamę ustna bądź nosową do przełyku w miejscu jego przylegania do lewego przedsionka. W momencie ustawienia elektrody w pobliżu lewego przedsionka podejmowana jest próba indukcji migotania przedsionków. U 90% pacjentów z zespołem WPW test przebiega pozytywnie i wyzwala się migotanie przedsionków [11],
- Stymulacja serca przez dozylną droge centralną: tametoda jest uważana za najbardziej efektywną w ocenie ryzyka wszystkich pacjentów z WPW niezależnie od objawów. Aby przeprowadzić to badanie należy wprowadzić cewnik z elektrodą dożylnie bezpośrednio do lewego przedsionka, który stymulujemy by wywołać migotanie przedsionków [11].

Ponadto do diagnostyki wykorzystuje się badania genetyczne, które powinny być wykonywane u pacjentów z obciążającym wywiadem. Polegają one głównie na molekularnej analizie genetycznej metodami PCR i dHLPC. Najczęściej wykrywana jest insercja lub delecje w genie 7q3 kodującym filaminę znajdującą się mięśniu sercowym. W Polsce badania tego rodzaju są mało dostępne [1].

U 25-55% pacjentów z dodatkowymi drogami przewodzenia, nie ma widocznej w EKG fali delta w momencie, gdy rytm zatokowy ma zdolność przewodzenia impulsu tylko wstecznie z komory do przedsionka, wtedy określane jest to, jako ukryta droga dodatkowa. Lewostronne połączenie może nie być widoczne w EKG ze względu na jego preferencyjne przewodzenie przez węzeł przedsionkowo-komorowy, co może nie ujawnić się podczas wykonywania testów diagnostycznych [6].

## Leczenie

Odmienność w kontroli arytmii w zespołach preekscytacji u dzieci i dorosłych polega głównie na większej przydatności stymulacji nerwu błędnego u dzieci oraz szerszym użyciu środków farmakologicznych u dorosłych pacjentów [1]. Pierwszym krokiem podczas napadu AVRT u dzieci jest jego niefarmakologiczne przerwanie poprzez stymulację nerwu błędnego (próba Valsalvy, jednostronny masaż zatoki szyjnej, prowokacja odruchu wymiotnego, a u niemowląt metoda szoku termicznego, czyli położenie zimnego okładu na twarz lub okolicę przedsercową na 10-30s.). Jeżeli po pobudzeniu nerwu błędnego nie ma efektu należy podać adenozynę, która otwiera kanał potasowy oraz wapniowy powodując hiperpolaryzację błony komórkowej, a w efekcie zwalnia przewodnictwo w węźle przedsionkowo-komorowym i przerywa nawrotny częstoskurcz węzłowy [15]. W badaniach z 2000 roku u dzieci poniżej 18 roku życia z nawrotnym częstoskurczem nadkomorowym u 79% udało się przywrócić za pomocą adenozyny rytm zatokowy [15]. Należy ją podawać w szybkim wstrzyknięciu dożylnym (bolus), u niemowląt i małych dzieci w dawce 0,1 mg/ kg m.c./dawkę, a u starszych dzieci 3 mg/dawkę. Przy braku efektu zastosowanej terapii należy ponownie podać adenozynę po trzech minutach w dawce 2-krotnie większej od poprzedniej (u najmłodszych dzieci nawet do 0,3 mg/kg m.c./dawkę) [3, 15].

Inne leki mające zastosowanie w przerywaniu napadów AVRT to: 1) propafenon w bolusie co 10 minut 0,2-1 mg/ kg m.c/dawkę, następnie wlew iv. 4-7 mcg/kg m.c./min., 2) amiodaron w bolusie iv. w 5% glukozie przez 5-10 min. 5 mg/kg, następnie 1-2 h wlewy iv. lub 3) sotalol 0,2-1,5 mg/ kg m.c./dawkę we wlewie iv. przez 30-60 min. Adenozyna może być także przydatnym narzędziem diagnostycznym, ponieważ u pacjentów z niewielkim stopniem preekscytacji oraz ukrytą dodatkową drogą przewodzenia może ona pomóc uwidocznić preekscytację komór [3]. U dziecka niestabilnego hemodynamicznie, u którego brak jest dostępu donaczyniowego lub u którego leczenie farmakologiczne było nieskuteczne w przywróceniu rytmu zatokowego wskazana jest kardiowersja elektryczna (zsynchronizowana z załamkiem R). Pierwsza dawka energii dla kardiowersji w przypadku AVRT wynosi 0,5 –1 J/kg m.c, a druga 2 J/kg m.c. Jeżeli kardiowersja była nieskuteczna, przed podjęciem trzeciej próby należy podać amiodaron lub prokainamid [16]. W przypadku opornego na leczenie AVRT skuteczna okazać się może dożylna podaż prokainamidu lub amiodaronu, przy czym według danych z piśmiennictwa prokainamid okazuje się być skuteczniejszy [7, 10].

Terapia AVRT u pacjentów posiadających dodatkową drogę przewodzenia zależna jest od wieku pacjenta, klinicznych objawów preekscytacji oraz towarzyszących strukturalnych chorób serca. Celem terapii jest zapobieganie nawrotom AVRT, a także zmniejszenie ryzyka poważnych zaburzeń rytmu, w tym migotania komór lub nagłego zatrzymana krążenia. Istnieją dwie podstawowe opcje terapeutyczne: zachowawcza terapia farmakologiczna oraz inwazyjna - ablacja.

W leczeniu przewlekłym, zabezpieczającym pacjenta przed napadami częstoskurczu leki antyarytmiczne, głównie doustne preparaty beta- blokerów (II klasa leków antyarytmicznych), są zwykle lekami pierwszego rzutu. Nie wpływają one bezpośrednio na przewodnictwo przez dodatkową drogę, ale ograniczają epizody AVRT, które mogą wywołać migotanie przedsionków, przez co pośrednio mogą one zmniejszyć ryzyko nagłego zgonu [15]. Beta – blokery, głównie propranolol i metoprolol, hamują wpływ układu współczulnego na aktywność elektryczną serca, spowalniają zatokowy rytm serca i szybkość przewodzenia. Doustnie propranolol dawkuje się początkowo 0,5-1 mg/kg m.c./dobę w 3-4 dawkach podzielonych, dawka podtrzymująca wynosi 2-4 mg/kg m.c./dobę w 3-4 dawkach podzielonych [15]. Escudero i wsp. podają też zastosowanie innych leków, jak np. prokainamid - lek antyarytmiczny klasy IA umiarkowanie wydłużający czas repolaryzacji [15]. Flekainid (lek antyarytmiczny klasy IC) niektórzy autorzy zalecają, jako lek pierwszego rzutu w prenatalnym leczeniu płodowego AVRT. Kolejny lek tej grupy, propafenon, oprócz blokowania kanałów sodowych posiada także działanie β- adrenolityczne, przez co wydłuża okres refrakcji w przedsionkach i komorach, jak też spowalnia przewodnictwo w węźle przedsionkowo – komorowym. Może być podawany doustnie lub dożylnie w dawce 8-9 mg/ kg m.c./dobę w 4 dawkach podzielonych, a następnie w 3 dawkach podzielonych [15]. Opisuje się też zastosowanie Amiodaronu (III klasa leków antyarytmicznych). Blokuje on kanały potasowe, wydłuża czas repolaryzacji i przedłuża okres refrakcji dla mięśnia przedsionków i komór oraz zmniejsza przewodnictwo w węźle zatokowym oraz węźle przedsionkowo-komorowym. Podawany jest on w dawkach: I doba – 15 mg/kg m.c., II doba – 10 mg/kg m.c. i od III doby – 5 mg/kg m.c. Według badań amiodaron pozwalał na kontrolowanie rytmu serca już u niemowląt oraz dzieci z WPW manifestującym się AVRT. Amiodaron stosowany jest u pacjentów z AVRT, którzy przyjmują także beta-blokery [15]. W badaniach Price’a i wsp. kombinacja flekainidu i sotalolu pozwoliła na skuteczne farmakologiczne kontrolowanie AVRT u niemowląt [17]. U dzieci, u których pojawił się epizod napadu częstoskurczu okresowa terapia farmakologiczna lub nauka stymulacji nerwu błędnego zwykle są skuteczne, jeśli są to pacjenci niskiego ryzyka [7]. Należy unikać stosowania digoksyny lub blokerów kanału wapniowego ze względu na ich zdolność do skrócenia okresu refrakcji dodatkowej drogi przewodzenia, a nawet przyspieszania przewodnictwa w tej drodze, co może doprowadzić do migotania przedsionków [6,15].

Za najbardziej efektywną metodę leczenia zespołów preekscytacji uznana jest ablacja dodatkowej drogi przewodzenia. Jest ona skutecznym zabiegiem dla objawowych pacjentów zagrożonych nagłym zgonem sercowym, którzy są w odpowiednim wieku [11]. Polega ona na przerwaniu dodatkowej drogi przewodzenia w sercu za pomocą elektrody wprowadzanej przez tętnicę lub żyłę udową do lewego przedsionka [18]. Przed zabiegiem ablacji należy zlokalizować położenie dodatkowej drogi przewodzenia, czyli wykonać tzw. mapowanie. Mapowanie przeprowadza się na podstawie kryteriów anatomicznych oraz elektrofizjologicznych opierając się na zapisach z elektrod ablacyjnych [19]. Oprócz badań elektrofizjologicznych przydatne jest tzw. mapowanie elektroanatomiczne (np. system CARTO), które pozwala na bezpośrednią rejestrację potencjałów wewnątrzsercowych w odniesieniu do anatomii jam serca. Po zlokalizowaniu dodatkowej drogi przewodzenia istnieje kilka metod ablacji [20]. Rozgrzewając końcówkę elektrody prądem o częstotliwości radiowej (do ok. 60 stopni Celsjusza) wykonuje się tzw. RF ablację (radio frequency ablation), natomiast posługując się niską temperaturą (do ok. -70 stopni Celsjusza) wykonuje się tzw. krioablację [21]. Aktualne wytyczne American Heart Association, American College of Cardiology i European Society of Cardiology ograniczają ablację dla bezobjawowych pacjentów z zespołem WPW tylko do osób z zawodami wysokiego ryzyka i zawodowych sportowców [13]. Ablacja powinna być wykonana u dzieci dopiero powyżej 10 roku życia z obawy na wytworzenie relatywnie dużej blizny pozabiegowej w stosunku do rozmiaru serca, jednak zazwyczaj przeprowadza się ten zabieg u dzieci ważących powyżej 15 kg [7]. U pacjentów z AVRT związanym z zespołem preekscytacji w wieku powyżej 5 lat u ponad 75% występują nawroty częstoskurczu, dlatego też większość placówek oferuje tej grupie pacjentów zabieg ablacji, jako formę terapii pierwszorzędowej. Wytyczne dotyczące momentu, w którym można brać pod uwagę przeprowadzenie takiej interwencji opiera się na ocenie ryzyka, umiejscowieniu dodatkowej drogi przewodzenia oraz częstości występowania i nasilenia objawów. Efektywność ablacji u dzieci jest bardzo wysoka, wynosi ona dla lewej wolnej ściany serca 97-98%, prawej wolnej ściany 91-95%, przedniej części przegrody 83-86% oraz tylnej część przegrody 93%. Nawrót częstoskurczu po skutecznej ablacji drogi dodatkowej u młodych pacjentów szacowany jest na około 11% i zwykle występuje w ciągu pierwszych 2 miesięcy po zabiegu. Najczęściej nawroty wystąpiły u dzieci posiadających dodatkową strukturalną wadę serca oraz posiadających więcej niż jedną dodatkową drogę przewodzenia [13]. W badaniu przeprowadzonym przez Blaufoxa i wsp. nie odnotowano istotnych różnic w powikłaniach u dzieci w wieku poniżej 18 miesięcy poddanych ablacji w porównaniu do starszych, ale dzieci z dodatkowymi połączeniami w przegrodzie miały istotnie większe ryzyko powikłań w stosunku do pacjentów z dodatkowymi drogami umiejscowionymi w innych częściach serca [6]. Ablacja chirurgiczna aktualnie brana jest pod uwagę u pacjentów z anomalią Ebsteina i dodatkowymi drogami podlegającymi wewnątrzsercowej naprawie oraz u innych pacjentów ze złożonymi wadami wrodzonymi, które leczone są chirurgicznie [6]. Krioablacja jest szeroko stosowana w praktyce pediatrycznej ze względu na większe bezpieczeństwo zabiegu oraz mniejsze ryzyko powstania w sercu blizny po ablacji, szczególnie u osób z drogami dodatkowymi w okolicach przegrody lub w bliskim sąsiedztwie zatoki wieńcowej [13].

Poważne działania niepożądane przypisywane ablacji to blok przedsionkowo-komorowy, perforacja serca, perforacja tętnicy wieńcowej oraz powikłania zakrzepowo-zatorowe. Zgony odnotowywane, jako powikłanie pediatrycznych ablacji nastąpiły wskutek perforacji serca, urazu mięśnia sercowego, zakrzepów wieńcowych lub mózgowych i komorowych zaburzeń rytmu serca. U pediatrycznych pacjentów odsetek zgonów po ablacji wynosi 0.22% [13].

## Zespół wpw, a aktywność fizyczna

W badaniu opisującym przyczyny nagłych zgonów sercowych u sportowców, zespół WPW odpowiedzialny był za około 1% NZK. W Polsce zgodnie z Rozporządzeniem Ministra Zdrowia z dnia 22 lipca 2016, każde dziecko przed otrzymaniem orzeczenia o zdolności do uprawiania określonej dyscypliny sportowej musi mieć wykonany m.in. zapis EKG. W ostatnich latach zwiększenie liczby wykonywanych spoczynkowych zapisów EKG, jako skrining przed dopuszczeniem do zajęć sportowych, doprowadziło do zwiększonej rozpoznawalności asymptomatycznych pacjentów z preekscytacją o typie WPW. Optymalne leczenie tej grupy pacjentów ciągle pozostaje problematyczne. Podczas gdy udany zabieg ablacji eliminuje ryzyko nagłego zgonu sercowego u bezobjawowych pacjentów z cechami preekscytacji w zapisie EKG, to kierowanie każdego pacjenta na ten zabieg może nieść za sobą również większe ryzyko powikłań. Wstępna ocena uczestnictwa bezobjawowych pacjentów pediatrycznych w zajęciach sportowych, włączając zajęcia WF, powinna zawierać wykonanie zapisu spoczynkowego EKG [13]. U każdego pacjenta pediatrycznego z rozpoznanymi cechami preekscytacji w zapisie EKG, który ma być dopuszczony do uprawiania sportu należy ocenić zapis EKG (każdy zespół QRS) pod kątem stałości utrzymywania się tych cech. Jeśli cechy preekscytacji występują w spoczynkowym zapisie EKG na stałe, należy skierować pacjenta na dalsze badania pozwalające ocenić czynniki ryzyka nagłego zgonu sercowego. Zazwyczaj dodatkowo oceniany jest 24-godzinny zapis EKG metodą Holtera, próba wysiłkowa oraz badanie echokardiograficzne, które pozwala wykluczyć strukturalne choroby serca, takie jak zespół Ebsteina czy kardiomiopatię przerostową. Pacjenci nieuprawiający wyczynowo sportów, a także sportowcy biorący udział w sportach o niskim natężeniu wysiłku fizycznego, u których w trakcie zapisu EKG metodą Holtera lub podczas próby wysiłkowej rejestruje się wyraźną utratę cech preekscytacji, mogą być zaliczeni do grupy niskiego ryzyka wystąpienia zagrażającej życiu arytmii i mogą być jedynie edukowani w zakresie niepokojących objawów, na które powinni zwracać uwagę oraz poddawani okresowym badaniom kontrolnym. Jednak w sportach o umiarkowanym i wysokim natężeniu wysiłku fizycznego sportowcy z intermitującym zespołem WPW lub, u których na szczycie wysiłku dochodzi do wygaszenia cech preekscytacji, powinni być poddani dalszej stratyfikacji ryzyka za pomocą badań elektrofizjologicznych [22]. Zgodnie z ustaleniami 36 Bethesda Conference konieczna jest dokładna ocena ryzyka, która powinna obejmować również przeprowadzenie badań elektrofizjologicznych u bezobjawowych sportowców, którzy poddawani są umiarkowanemu lub intensywnemu wysiłkowi fizycznemu, szczególnie w sportach wyczynowych. Stanowisko European Society of Cardiology jest jednak nieco bardziej rygorystyczne, nakłada ono bowiem obowiązek przeprowadzenia gruntownej oceny ryzyka nagłej śmierci sercowej, włącznie z wykonaniem badania elektrofizjologicznego u każdego sportowca z zespołem WPW, niezależnie od stopnia wykonywanego wysiłku fizycznego. Czynniki stanowiące podwyższone ryzyko nagłego zgonu w badaniu elektrofizjologicznym to: krótki czas refrakcji drogi dodatkowej (SPERRI) <240 ms lub <220 ms po podaniu izoprenaliny, obecność mnogich dodatkowych dróg przewodzenia lub łatwo indukowane migotanie przedsionków. Podczas gdy ablacja jest zalecaną formą terapii u bezobjawowych pacjentów z zespołem WPW, dla tych pacjentów, którzy nie zgadzają się na zabieg lub u których dodatkowa droga przewodzenia zlokalizowana jest w okolicy pęczka Hisa, wyczynowe uprawianie sportu może być dozwolone, jeśli żaden z wyżej wymienionych czynników ryzyka nie wystąpi w badaniu elektrofizjologicznym. Pomimo różnic w wytycznych amerykańskich i europejskich, każde dziecko z zespołem preekscytacji niezależnie od tego czy uprawia sport, powinno zostać skierowane na szczegółowe badania oceniające czynniki ryzyka nagłego zgonu sercowego do ośrodka specjalizującego się w badaniach elektrofizjologicznych u pacjentów pediatrycznych [13].

Podsumowując, zespoły preekscytacji, w szczególności zespół WPW, jest istotnym problemem zwłaszcza w populacji dzieci i młodzieży, które uprawiają sport czy też wybierają przyszły zawód. Ważne jest wczesne wykrywanie zespołu WPW, które umożliwia kontrolowanie częstoskurczu oraz zabezpiecza przed groźnymi dla życia arytmiami. W dzisiejszych czasach jest to szczególnie ważne ze względu na powiększającą się liczbę ośrodków pediatrycznych wykonujących zabieg ablacji, który daje szansę na normalne życie dzieci z tym istotnym problemem zdrowotnym.
